# Peripheral nervous system involvement in SARS-CoV-2 infection: a review of the current pediatric literature

**DOI:** 10.3389/fneur.2023.1134507

**Published:** 2023-05-25

**Authors:** Lorenzo Perilli, Marina Fetta, Martina Capponi, Cristiana Alessia Guido, Salvatore Grosso, Paola Iannetti, Alberto Spalice

**Affiliations:** ^1^Department of Molecular Medicine and Development, University of Siena, Siena, Italy; ^2^Department of Maternal, Infantile, and Urological Sciences, Faculty of Medicine and Dentistry, Sapienza University of Rome, Rome, Lazio, Italy

**Keywords:** SARS-CoV-2, peripheral nervous system diseases, children, complication, cranial neuropathies, Guillain-Barré syndrome, Miller Fisher syndrome

## Abstract

Severe acute respiratory syndrome coronavirus 2 (SARS-CoV-2) was identified as the pathogen responsible for the pandemic health emergency declared by the World Health Organization in March 2020. During the first part of the pandemic, adults showed mild to severe respiratory symptoms. Children seemed initially exempt, both from acute and subsequent complications. Hyposmia or anosmia were promptly identified as the main symptoms of acute infection, so neurotropism of SARS-CoV-2 was immediately suspected. (1, 2). As the emergency progressed, post infectious neurological complications were described also in pediatric population (3). Cases of cranial neuropathy in connection with acute SARS-CoV-2 infection have been reported in pediatric patients, as an isolate post infectious complication or in the context of the multisystem inflammatory syndrome in children (MIS-C) (4–6). Neuroinflammation is thought to be caused by several mechanisms, among which immune/autoimmune reactions (7), but so far, no specific autoantibody has been identified. SARS-CoV-2 can enter the central nervous system (CNS) directly and/or infect it retrogradely, through the peripheral nervous system (PNS), after replicating peripherally; several factors regulate invasion and subsequent neuroinflammation. Indeed, direct/secondary entry and replication can activate CNS-resident immune cells that, together with peripheral leukocytes, induce an immune response and promote neuroinflammation. In addition, as we will discuss in the following review, many cases of peripheral neuropathy (cranial and non-cranial) have been reported during or after SARS-CoV-2 infection. However, some authors have pointed out that the increase of cranial roots and ganglia in neurological imaging is not always observed in children with cranial neuropathy. (8). Even if a variety of case reports were published, opinions about an increased incidence of such neurologic diseases, linked to SARS-CoV-2 infection, are still controversial (9–11). Facial nerve palsy, ocular movements abnormalities and vestibular alterations are among the most reported issues in pediatric population (3–5). Moreover, an increased screen exposure imposed by social distancing led to acute oculomotion’s disturbance in children, not primarily caused by neuritis (12, 13). The aim of this review is to suggest food for thought on the role of SARS-CoV-2 in neurological conditions, affecting the peripheral nervous system to optimize the management and care of pediatric patients.

## Introduction

SARS-CoV-2 is a beta-coronavirus with a 29,903 base single stranded RNA genome that belongs to the Coronaviridae family. At the beginning of the health emergency, it was thought that SARS-CoV-2 was putative only for a severe respiratory distress syndrome, mostly observed in adult population. In addition to the main involvement of the respiratory tract, various associated symptoms were recognized as primary features of acute infection, such as anosmia and loss of taste. In consideration of the observed neurotropism, various mechanisms of neuro-invasion were hypothesized. In 2020 Lin et al. described a cohort of patients presenting post-infectious complications: 8.9% of the patients reported symptoms involving cranial nerves, hypogeusia, hyposmia, hypopsia, and neuralgia (14). In adults, the pathophysiology of COVID-19 has been hypothized strictly related to a cytokinic storm, mainly responsible for cardiovascular complications. As the pandemic progressed, cases of multisystem inflammatory syndrome in children (MIS-C) (15) and Miller Fisher syndrome (MFS) were reported, both in adults and children (16, 17). At the very beginning, children were considered exempt from both acute infection and post-infectious sequelae; in fact, most of the children showed few symptoms or were completely asymptomatic. Symptoms involving the peripheral nervous system, particularly facial nerve function, were among the first observed during acute infection in children (9, 18). Numerous manuscripts have been published on neurological involvement in SARS-CoV-2 infection. However, epidemiological data are often biased and inconsistent, partly due to the rapid evolution of virulence and pandemic scenarios. There is a need to further investigate the actual incidence of cranial neuropathy related to SARS-CoV-2 infection in the pediatric population, either alone or associated with MIS-C.

## Methods

Relevant studies were systematically searched by the authors in electronic databases, including MEDLINE and especially PubMed. Data were extracted from articles reporting pediatric cases of peripheral nervous system involvement in certain or probable SARS-CoV-2 infection. A search strategy based on keyword association was used: (“COVID-19” or “2019-nCoV” or “SARS-CoV-2”) and (“peripheral nervous system” or “neurotropism” or “cranial nerves” or “Guillain-Barré” or “MIS-C”). Cases were collected through analysis of case reports and case series published from the beginning of the pandemic in 2020 to the present. In addition, numerous reviews were used to support the data collected. Although we conducted large-scale research, we focused exclusively on pediatric cases.

## Discussion

### SARS-CoV-2 neurotropism

SARS-CoV-2 infection has been described in the literature as causing several neurological symptoms involving the central and peripheral nervous system. Different pathogenetic mechanisms and different mechanisms of access to the nervous system have been suggested. Even if the central nervous system (CNS) is structured to be protected by most of the infections, as observed for other viruses, SARS-CoV-2 is thought to be able to enter through hematogenous routes or neuronal retrograde routes. Through the hematogenous route the virus infects the blood–brain-barrier or the cells of the choroid plexi and subsequently assaults the neuronal tissue and activates the microglia in defense of the CNS (19). Similar to other viruses, it has been hypothesized that SARS-CoV-2 can use trans-synaptic retrograde transport from the peripheral nervous system as a pathway of infection to the brain. (20). Moreover, latest molecular studies evidence that human angiotensin converting enzyme 2 (hACE2) receptor, transmembrane serine protease 2 (TMPRSS2) and basigin (BSG, or CD147) are active factors involved in these routes (21). SARS-CoV-2, as a coronavirus, can bind the ACE2 receptor to enter human cells; for this reason, given the expression of ACE2 on neurons and glial cells, including the olfactory bulb, it is plausible that they may be targets of the virus to penetrate the host. (2) A transcriptional pathway that allows SARS-CoV-2 to access the CNS through the olfactory nerve, piriform cortex, and interaction with the ACE2 receptor has also been described (22). On the other hand, some authors have speculated a role of reduced ACEr expression in children as a possible explanation for the low susceptibility of children to the neurological complications of SARS-CoV-2 infection (23). In addition to these hypotheses, some authors argue that the neurological symptoms of SARS-CoV-2 infection are caused by a systemic inflammatory response. The resulting cytokine storm affects the blood–brain barrier, either directly or through an autoimmune response, as in the Guillain-Barré syndrome model (24). Therefore, an immune-mediated mechanism in acute and post-infectious neurological sequelae is also plausible in the reported peripheral nervous system manifestations. However, given the high prevalence of SARS-CoV-2 infection, it is difficult to establish causal links.

### Cranial nerves involvement in SARS-CoV-2 infection

Cranial nerves are part of the peripheral nervous system (PNS); however, the first and second cranial nerves (olfactory and optic nerves, respectively) are considered to be extensions of the central nervous system. The nuclei of cranial nerves are in the central nervous system (CNS). Through the axons, stimuli are conducted to the PNS and from there return to the CNS. The cranial nerves mentioned in this review are olfactory (I), optic (II), oculomotor (III), abducens (VI), facial (VII) and vestibulocochlear (VIII).

We present below the cases, reported in the literature between 2019 and 2022, of their acute and post-infectious involvement in SARS-CoV-2 infection in the pediatric population. For a more usable discussion, we report them in numerical order, starting with the first cranial nerve.

### I cranial nerve

Olfactory nerve involvement in SARS-CoV-2 infection is described in pediatric patients presenting with hypo/anosmia. However, the only pediatric patient reported in the literature who presented with this symptom did not show nerve I enhancement on brain MRI. As reported by other authors, a cranial nerve dysfunction does not always find a neuroradiological correlate (8).

### II cranial nerve

The second cranial nerve is more properly part of the central nervous system. Several cases of optic neuritis related to SARS-CoV-2 infection have been reported. The association between optic neuritis and various viral infections is well known. Unilateral optic neuritis was described as an initial presentation of SARS-CoV-2 infection in a 10-year-old girl with vision loss in her left eye but without typical symptoms of COVID-19 (25). Another case presented with bilateral optic neuritis in neuromyelitis optica spectrum disorder (NMOSD), 10 days after a febrile illness highly suspected as SARS-CoV-2 infection (both parents were positive) (26). In addition, a case of a 13-year-old boy was reported who presented with symptoms and MRI indicative of optic neuritis. Cerebrospinal fluid (CSF) analysis was nondiagnostic, but serologic test for SARS-CoV-2 was positive; therefore, the patient was labeled as affected by post-infectious optic neuritis. Additionally, two adolescents with optic neuritis (bilateral and unilateral), clinically and serologically associated with SARS-CoV-2, were reported. (27) All of these patients had no history of neurological conditions.

### III cranial nerve

De Oliveira et al. reported the case of a 2-year-old girl who presented with acute divergent strabismus and ptosis of the right eye. The patient had no radiological signs consistent with the clinical picture. In the absence of any other explanation, given the IgM positivity for SARS-CoV-2, III nerve palsy caused by SARS-CoV-2 was diagnosed. Ophthalmologic examination showed ptosis of the upper eyelid with visual axis impairment and anisocoria with discrete mydriasis in the right eye. Interestingly, this is the first case report linking acquired nonpupillary oculomotor nerve palsy to asymptomatic acute SARS-CoV-2 infection in a child. (28) Lonardi et al. reported the case of a 2-year-old girl with sphingosine phosphate lyase insufficiency syndrome, who developed MIS-C 3 weeks after SARS-CoV-2 infection, complicating in unilateral right eye ophthalmoplegia, ptosis, mydriasis, consistent with a III nerve palsy. The child came to the attention of pediatricians 15 days after the onset of paralysis and showed no additional neurological symptoms beyond those previously attributed to her syndrome. The patient was treated with prednisone for 5 days; after 1 month, follow-up examination showed no complete resolution of the III nerve palsy (29).

### VI cranial nerve

Capponi et al. described the case of a 9-year-old girl with abducens nerve palsy following SARS-CoV-2 infection. The complication appeared about 2 weeks after the infection, which occurred paucisymptomatically. The child then presented with an acute form of convergent strabismus of the right eye and headache without meningeal signs. Other than that, the neurological examination was normal. In addition, lumbar puncture and blood tests showed normal parameters. Therefore, abducens palsy probably related to previous SARS-CoV-2 infection was clinically diagnosed (30). Chung et al. described the case of a bilateral papilledema associated with abducens nerve palsy in a 12-year-old child already affected by myopia, astigmatism, and mild amblyopia. At the onset the child showed fever, vomit, diarrhea associated with fatigue, dysgeusia, photophobia and two episodes of nose bleeding. Parents reported previous symptoms compatible with SARS-CoV-2 infection, but no tests were performed. The patient was admitted subsequently for persistent intermittent fever and diarrhea, presenting with increased inflammation markers and positive IgG for SARS-CoV-2. During the recovery the clinical picture complicated in a MIS-C. One week into admission, the patient complained of intermittent episodes of headache accompanied by bilateral blurred vision and binocular diplopia. The patient was dismissed after 12 days and at follow up examination, 2 months later, showed complete resolution (31).

Moreover, Costagliola et al. reported three cases of neurovisual manifestations in children with SARS-CoV-2 infection; the first case is a previously healthy 2-year-old boy affected by a monocular convergent strabismus of the right eye occurred after 2 days of fever, in lack of any other neurological findings, PCR for SARS-CoV-2 resulted positive and an ophthalmological evaluation that did not find any abnormalities. MRI of the brain and orbits was normal. One week after onset, the child recovered but developed a left convergent strabismus that persisted at 2-month follow-up with loss of stereopsis. The second case was a 5-year-old child who developed an isolated convergent monocular strabismus of the right eye with diplopia 10 days after SARS-CoV-2 infection, which resolved after 24 h; no recurrences were reported at 2 months follow-up. The third case is an 8-year-old boy who developed, after SARS-CoV-2 infection, reduction of visual acuity and perception: the child reported seeing objects as bigger or smaller than usual. This condition persisted 1 month after the onset and resolved at 1-month follow up. This is the first reported case of Alice in Wonderland Syndrome associated to SARS-CoV-2 infection (32).

### VII cranial nerve

The pathogenesis of peripheral facial nerve palsy (FNP) is poorly understood. In the pediatric population, the most common cause of FNP (60-80% of cases) is Bell’s palsy, the idiopathic form. The remaining cases are secondary to infectious diseases, neoplasms, trauma, and congenital abnormalities. Prominent among infectious causes are neurotropic germs, particularly viruses. It is hypothesized that viral replication and spread in the axon may lead to demyelination and inflammation (33). Sequently unilateral palsy of the muscles innerved by facial nerve is observed. Symptoms usually regress in weeks or months, but sometimes become permanent (9), and are reported in the acute stage of infection or in the first week after the onset of viral symptoms (33). In United Kingdom some authors pointed out that during the first part of the pandemic was observed an increased number of FNPs (9); differently others had not found a causal link between FNP and the virus, more an increased incidence of FNP in a high prevalence of the infection (10). On the other hand, a retrospective study conducted by Andina-Martinez *et Al* showed an increased incidence of FNP during 2020–2021 in a high prevalence area of infection in Spain (18). Barron et al. reported the results of a retrospective study about the increased incidence of a SARS-CoV-2 infection-related FNP studying the patients admitted between 2015 and 2020 in two British hospitals, including children affected by isolated FNP (10). The limitation to this study is the restricted number of patients and the bias imposed by the co-presence of new viral variants. On the other hand, in the same period, different authors described a statistically significant (*p* < 0.0001) increase in the number of SARS-CoV-2 infection-related facial nerve palsies in the period February–June 2020 (9). Andina-Martinez *et Al* retrospectively analyzed the incidence of Bell’s palsy before and during the pandemic: 29 patients were admitted during the first year of pandemic in the Hospital Infantil Niño Jesús de Madrid presenting with facial nerve palsy. A high number of them tested positive for SARS-CoV-2 IgG. In the 3 years before the health emergency was declared, only 24 patients were registered for Bell’s palsy. The authors underline how difficult is to link SARS-CoV-2 to facial neve palsy, given the variety of viral infections that can lead to this complication (18). In addition to that Al Ozonoff et al. reported that the percentage of facial nerve palsy post vaccination against SARS-CoV-2, is consistent with those expected in general population (11).

The first case of FNP associated with SARS-CoV-2 infection in a male child presenting with right facial nerve palsy and difficulty closing the eye, exacerbated 10 days after SARS-CoV-2 infection, decurred with mild symptoms, was reported. On examination, he showed signs of right FNP with resting asymmetry, incomplete closure of the right eye (4/5 of eyelid excursion, with Bell’s sign), and absence of active movements in the frontal, superciliary corrugator, nasal, zygomatic, and right risorius muscles, with mild activation of the quadratus labii inferioris muscle. The rest of cranial nerve tests were normal, neurological examination was unremarkable. Blood tests showed normal parameters. He performed neuro-muscular rehabilitation 3 times a week. After 6 months, the clinical picture was not completely resolved. MRI showed no nerve enhancement or thickening. (34) Hookham et al. described the case of a 17-year-old girl affected by MIS-C (also known as PIMS-TS): after 16 days from the onset of the acute infection she presented facial nerve palsy (4). Zain et al. described the case of a 23-month-old patient presenting acute facial palsy, tested positive for SARS-CoV-2, without other symptoms of infection. Brain MRI showed enhancement in signal of the canalicular segment of the right VII nerve, extending to the first genu, suggesting signs of neuritis. No other clinical symptoms or altered parameters in blood tests were observed. The patient was successfully treated with steroid therapy (35). Iacono et al. reported the case of a 5-year-old child affected by a fourth degree House-Brackmann grading system (obvious facial weakness, incomplete eye closure, no forehead movement, asymmetrical mouth movement, and synkinesis) facial palsy. Blood tests showed positive IgG for SARS-CoV-2. No other significant parameters or criteria compatible with MIS-C were found (36). Teophanus et al. described the case of a 6-year-old boy affected by FNP during acute SARS-CoV-2 infection. The child, previously diagnosed with hyper-IgM syndrome, presented with facial palsy, inability to close the right eye and right sided mouth droop with drooling (House-Brackmann grade: IV) (37).

### VIII cranial nerve

Vestibular neuritis (VN), defined as a benign, self-limited vestibular imbalance, is one of the most common causes of vertigo in children, especially among adolescents, and accounts for about 16% of overall pediatric vertigo. Giannantonio et al. described vestibular nerve involvement as a peripheral neurological complication related to SARS-CoV-2 infection. The case of a 13-year-old boy who tested positive for SARS-CoV-2 and suffered from fever, dizziness, and repeated vomiting was reported. In addition, a vestibular examination performed by an otolaryngologist showed the presence of a grade III spontaneous horizontal-torsional nystagmus with a fast rightward, nonrhythmic, inexhaustible component of about 70 bpm, also visible with visual fixation, although weaker. No neuroimaging changes were reported. The symptom disappeared 1 month after discharge with complete resolution. In view of the low prevalence of balance disorder in children and its known presence in post-infectious pictures, SARS-CoV-2 was considered the infectious trigger of the child’s clinical picture (38).

### Cranial nerve palsy in MIS-C

SARS-CoV-2 infection usually involves a moderate course in children. More rarely, severe complications may occur during acute infection, possibly with associated phenomena, as occurs in multisystem inflammatory syndrome in children (MIS-C) (39). Among neurological phenomena, headache is the most frequently described neurological symptom in SARS-CoV-2 infection (14). “Multisystem inflammatory syndrome in children” (MIS-C) is also known as “pediatric inflammatory multisystem syndrome temporally associated with SARS-CoV-2” (PIMS-TS). It is a rare condition that can occur 2–6 weeks after acute infection in children resulting in a cytokine storm that can also leads to multiorgan failure. Ongoing fever, stomach pain, bloodshot eyes, diarrhea, dizziness or lightheadedness, skin rash and vomiting occurring in a child with a suggestive anamnesis of SARS-CoV-2 infection, must alert parents and practitioners. There is no definite diagnostic test for MIS-C; therefore, a definition of MIS-C has been developed by the Royal College of Pediatrics and Child Health (RCPCH), the World Health Organization (WHO) and by the Centers for Disease Control and Prevention (CDC). All the three definitions include, in association with SARS-CoV-2 infection, fever for at least 24 h, laboratory signs of inflammation, a clinically severe condition with multiple organ damage (≥ 2), and no plausible alternative diagnosis (40). In a United Kingdom case-series study, out of 27 children with MIS-C, 4 had newly onset neurological symptoms, including encephalopathy, dysarthria, dysphagia, cerebellar ataxia and peripheral neuropathy leading to global proximal muscle weakness and reduced reflexes. Involvement of the peripheral nervous system was observed in all patients, with overall proximal muscle weakness and reduced reflexes. Neurological symptoms were part of the initial presentation in 2 patients (41) As we previously reported above, Lonardi et al. described a case of a 2-year-old girl suffering from MIS-C with a clinical picture of paralysis of the third cranial nerve (29). Hookham et al. reported the case of a 17-year-old girl with MIS-C and facial nerve palsy (4). Multisystemic inflammatory syndrome in children has been also associated with idiopathic intracranial hypertension, with ophthalmic symptoms as reported by Verkuil et al. A 14-year-old girl was admitted with fever, headache, rash, diarrhea, and dyspnea. Chest Computed tomography (CT) showed diffuse ground glass pattern and echocardiogram detected diffuse dilation of the right coronary artery. Her examination revealed also bilateral papilledema and brain MRI was consistent with elevated intracranial pressure. Serology proved evidence of antibodies versus SARS-CoV-2 and diagnosis of MIS-C was confirmed (42). Baccarella et al. reported two cases of headache and unilateral paralysis of the abducent nerve in MIS-C. The first, is a 9-year-old child who did not show papilledema but high opening pressure at lumbar puncture (LP), consistent with intracranial hypertension. The second patient is a 6-year-old child, who also showed bilateral papilledema. Brain and orbits MRI supported the diagnosis, however, the lumbar puncture showed normal opening pressure (43). Acetazolamide and steroid have been shown to be effective in all the mentioned cases of intracranial hypertension in MIS-C. Based on the reported literature, we believe that if MIS-C is suspected, as part of the diagnostic pathway, it is advisable to also perform a fundus examination, or rather a brain MRI, to undertake appropriate therapy as soon as possible.

### SARS-CoV-2 and Guillain-Barré syndrome spectrum

Guillain-Barré Syndrome (GBS) is also known as acute demyelinating inflammatory polyradiculoneuropathy. It is an autoimmune syndrome, and the main hypothesized causes are infectious. Campylobacter Jejuni, a microaerophilic, nonfermentative Gram-negative organism, is responsible for 25-50% of Guillain-Barré syndrome cases. Other infectious agents implicated include Cytomegalovirus, Epstein-Barr virus, measles virus, Influenza A virus, Mycoplasma Pneumoniae, Enterovirus D68, and Zika virus (44, 45). The clinical spectrum of GBS includes the classic sensorimotor form, Miller Fisher syndrome, bilateral facial palsy with paresthesias, pure motor, pure sensory, paraparesis, pharyngeal-cervical-brachial variants, polyneuritis cranialis (GBS–Miller Fisher syndrome overlap), and Bickerstaff brainstem encephalitis (46). A single study described the case of a 3.5-year-old boy who presented with MIS-C promptly developing progressive ascending paralysis, bilateral facial weakness, right ptosis, and external ophthalmoplegia. Cerebrospinal fluid analysis and brain MRI suggested the diagnosis of GBS, which was confirmed by nerve conduction tests (47). Frank C.H.M et al. reported the case of a 15-year-old male patient who presented with frontal headache with retro-orbital pain, accompanied by fever, which evolved into weakness and pain in the lower limbs, which later spread to the upper limbs. The patient tested positive for SARS-CoV-2 infection. Electroneurography showed signs of acute motor axonal neuropathy (AMAN), a variant of GBS (17). Miller Fisher syndrome (MFS) is considered a variant of GBS. It is typically associated with dysfunction of cranial nerves three, four and six; it may present with facial palsy, ophthalmoplegia, areflexia or ataxia (48). Studies on adult population suggests that SARS-CoV-2 infection may cause peripheral nervous system involvement, even before the resolution of classic respiratory symptoms (49). Al Haboob et al. reported the case of an 11-year-old boy admitted to the emergency room for vomiting, headaches, blurred vision, dysarthria, dysphagia, balance disorders and respiratory failure. The child tested positive for SARS-CoV-2. Based on the clinical presentation, the radiological findings and the nerve conduction study, the diagnosis of Miller Fisher syndrome (MFS) with posterior reversible encephalopathy syndrome (PRES) in association with SARS-CoV-2 infection was made. In addition, the cerebrospinal fluid analysis confirmed the cytological-albumin dissociation (50). The exact pathogenesis of neurological damage related to SARS-CoV-2 infection is not yet known. But, based on the well-known molecular mimicry between microbial and neural antigens, the role of SARS-CoV-2 could act as a potential trigger of autoimmunity in GBS. Moreover, failure to detect SARS-CoV-2 in most cerebrospinal fluid (CSF) samples support the hypothesis of an immune mechanism, rather than a direct invasion.

## Conclusion

SARS-CoV-2, as the pathogen responsible for this health emergency, challenged the scientific community who originally needed to figure out how to treat COVID-19 in its classic pulmonary manifestation. Subsequently, it became clear that many other conditions had occurred during an acute SARS-CoV-2 infection or as a post-infectious complication. The whole scientific community has used as much strength as possible to understand the pathogenic mechanism of the disease, the methods of transmission of the virus, and infection control strategies. It is widely recognized that SARS-CoV-2 can affect the central nervous system as well as the peripheral nervous system. However, in the literature, there are only reviews that look at the adult population. But, as discussed above, a wide variety of neurological manifestations affecting the peripheral nervous system have been described in relation to SARS-CoV-2 infection in children. The mechanism of haematogenic or trans-neuronal dissemination should be responsible for the most common neurological symptoms developed by patients with COVID-19 (e.g., hypogeusia, hyposmia, headache, vertigo, and dizziness) (51, 52).

Among the studies cited in this article, 19 are case reports or case series published after the COVID-19 outbreak. They report cases of peripheral nervous system involvement in SARS-CoV-2 infection. Interestingly, of the 29 reported pediatric cases, almost 2/3 are male. The overall median age is 9 years. The figure does not change when considering only the male population, while it rises to 10.5 when considering the female population. The youngest patient reported is a 23-month-old girl, while the oldest is a 17-year-old boy.

Regarding the isolated involvement of cranial nerves, the most reported peripheral complication is isolated acute paralysis of the facial nerve, perhaps related to axonal diffusion and viral replication, resulting in inflammation and demyelination (9, 33). However, a large number of polyneuropathies (cranial and spinal) are reported in association with SARS-CoV-2 infection. Many of them fall under Guillain-Barré syndrome or Miller-Fisher syndrome, with albuminocytologic dissociation on CSF analysis.

Guillain-Barré syndrome (GBS) associated with SARS-CoV-2 infection appears similar to classic post-infectious GBS and is probably caused by the same immune-mediated pathogenic mechanisms (17, 48, 49). This condition should also be immediately suspected in pediatric patients with a known history of SARS-CoV-2 infection and compatible symptomatology in order to initiate treatment as soon as possible.

In MIS-C, overproduction of cytokines during acute infection induces an abnormal immune-mediated response, resulting in a hyperinflammatory syndrome affecting multiple organs. (53) Some authors have reported cases of cranial nerve palsy in pediatric patients with MIS-C, as well as increased intracranial pressure (7, 8). For these reasons, as part of the diagnostic pathway of MIS-C, brain imaging should also be performed to rule out neurological involvement. Currently, it is not known which of the proposed neurovirulence pathways predominates in children with SARS-CoV-2 infection-related neurological complications; in fact, each is hypothetical and largely unverified at present. Considering the few reported cases, it is also difficult to accurately assess the risk of neurological symptoms of SARS-CoV-2 infection in children. Characterization of neurological involvement in SARS-CoV-2 disease is further complicated by the frequent asymptomaticity of the virus in the acute phase of infection.

A summary table of the cases reported in the literature, cited in this review, is presented below (Table 1). As can be clearly observed, neurological manifestations associated with SARS-CoV-2 infection have been found in the pediatric population often in the absence of respiratory symptoms.

**Table 1:** Clinical data of patients with peripheral nervous system involvement in SARS-CoV-2 infection reported in current literature.

Authors and Title	Publication types	Sex	Age	SARS-CoV-2 infection-related symptoms	SARS-CoV-2 infection-related neurological complications	CSF*	MRI**	Treatment	Diagnosis
**4. Hookham L et al. 2021 Jun.**	Case Report	m	17	fever, diarrhoea, vomiting, intermittent chest pain.	right-sided facial palsy, headache; myalgia and lethargy.	NR^a^	BRAIN: minimal enhancement of the tympanic portion of the right VII cn^c^.	steroids, eye patch	MIS-C related facial nerve palsy
**5. Roussel A et al. 2021 Mar.**	Case Report	f	6	cerebral vasculopathy (sickle cell anemia).	bilateral facial palsy, left trigeminal hypoesthesia, voice alteration, swallowing impairment.	normal findings	BRAIN: enhancement of the intracranial and meatal segments of VII and XII cn^c^.	IVIG^b^, remdesevir, tocilizumab	SARS-CoV-2 related cranial polyneuropathy (V, VII, XII cn^c^)
**8. Lindan CE et al. 2021 Mar.**	Multicenter Study	m	6	NR^a^	bilateral facial palsy, respiratory failure (progressive weakness of inspiratory and expiratory muscles).	NR^a^	BRAIN: bilateral enhancement of VII, XII cn^c^.	IVIG^b^	SARS-CoV-2 related cranial polyneuropathy (VII, XII cn^c^)
		m	9	none	gait difficulty and lower limb weakness.	albuminocytologic dissociation	SPINE: enhancement and thickening of cauda equina and cervical spinal nerve roots.	IVIG^b^	SARS-CoV-2 related peripheral neuropathy
		f	16	diarrhea	crural paraesthesia and hypoesthesia progressing to T8 sensory level.	albuminocytologic dissociation	BRAIN: enhancement of III, VI-VIII cn^c^ SPINE: enhancement of cauda equina	Steroids; IVIG^b^	SARS-CoV-2 related peripheral neuropathy
		f	2	MIS-C syndrome	four limb weakness and bladder dysfunction.	NR^a^	BRAIN: normal findings. SPINE: enhancement and thickening of the cauda equina.	supportive measures in ICU^d^	MIS-C related peripheral neuropathy
		m	13	NR^a^	vertigo, partial deafness.	NR^a^	BRAIN: Enhancement of VIII cn^c^	steroids	SARS-CoV-2 related cranial neuropathy (VIII cn^c^)
		f	12	NR^a^	four limb weakness; brainstem dysfunction.	NR^a^	BRAIN: enhancement III, V, VII cn^c^ SPINE: enhancement cauda equina.	steroids	SARS-CoV-2 related peripheral neuropathy (Anti-GM2+) and brainstem dysfunction
**16. Khalifa M et al. 2020 Sep.**	Case Report	m	11	acute upper respiratory tract infection with low-grade fever.	lower limb weakness; tingling in legs and feet.	albuminocytologic dissociation	SPINE: enhancement of the cauda equina nerve roots.	IVIG^b^	SARS-CoV-2 related GBS^d^
**17. Frank CHM et al. 2021 Jul.**	Case Report	m	15	frontal headaches; retro-orbital pain.	progressive symmetrical four limb weakness, absent deep tendon reflexes.	normal findings	BRAIN - SPINE: normal findings	steroids, IVIG^b^	SARS-CoV-2 related GBS^d^
**27. Sánchez-Morales et al: 2021 Jul.**	Case Series	m	9	none	pain in lower limbs, ascendant weakness, hypotonia, diminished MSR in lower limbs.	albuminocytologic dissociation	NR^a^	NR^a^	SARS-CoV-2 related GBS^d^
		m	14	fever, rhinorrhea	paresthesia in feet, ascendant weakness, hypotonia, diminished MSR in lower limbs.	albuminocytologic dissociation	NR^a^	NR^a^	SARS-CoV-2 related GBS^d^
		f	12	NR^a^	dysphonia, hypotonia, ascendant weakness, diminished MSR in upper limbs, and absent MSR in lower limbs.	albuminocytologic dissociation	NR^a^	NR^a^	SARS-CoV-2 related GBS^d^
		f	15	NR^a^	diplopia, bilateral ocular pain, and diminished visual acuity, left VI cranial nerve paresis.	normal findings	NR^a^	NR^a^	SARS-CoV-2 related cranial neuropathy (VI cn^c^)
**28. de Oliveira MR et al. 2021 Jun.**	Case Report	f	2	none	acute-onset divergent strabismus and ptosis in the right eye	NR^a^	NR^a^	physiotherapy	SARS-CoV-2 related cranial polyneuropathy (II, VI cn^c^)
**29. Lonardi V et al. 2021 Jul**	Case Report	NR^a^	2	MIS-C	ophthalmoplegia with exotropia, ptosis, and mydriasis	albuminocytologic dissociation	BRAIN: normal findings.	steroids	SARS-CoV-2 related cranial neuropathy (III cn^c^)
**30. Capponi M et al. 2022 Jun.**	Case Report	f	9	fever, headache	esotropia with limitation of abduction in the right eye	NR^a^	BRAIN: normal findings.	none	SARS-CoV-2 related cranial neuropathy (VI cn^c^)
**32. Costagliola G et al. 2023 Feb.**	Case series	m	2	fever	bilateral esotropia	NR^a^	BRAIN: normal findings.	NR^a^	SARS-CoV-2 related cranial neuropathy (VI cn^c^).
		m	5	fever, vomiting	right esotropia	NR^a^	NR^a^	NR^a^	SARS-CoV-2 related cranial neuropathy (VI cn^c^)
		m	8	fever, headache, myalgia	diplopia, right eye exotropia, alteration of visual perception (perception of objects larger or smaller than normal)	NR^a^	BRAIN: normal findings.	NR^a^	SARS-CoV-2 related Alice in Wonderland Syndrome and cranial neuropathy (III cn^c^)
**33. Iacono A et al. 2022 May**	Case Report	m	5	none	right facial paresis (incomplete closure of the right eye and deviation of the buccal rim)	NR^a^	BRAIN: normal findings.	steroids	SARS-CoV-2 related facial nerve palsy
**35. Ferreira EF et al. 2022 Jan.**	Case Report	m	11	fever, headache, fatigue and cough	right facial paresis	NR^a^	BRAIN: right posterior insular-parietal-opercular (pre-existing lesion).	steroids	SARS-CoV-2 related facial nerve palsy
**36. Zain S. et al. 2021 Jun.**	Case Report	f	2	none	right facial paresis	normal findings	BRAIN: enhancement of the right VII cn^c^.	steroids	SARS-CoV-2 related facial nerve palsy
**37. Theophanous C. et al. 2021 Feb.**	Case Report	m	6	none	right facial paresis	NR^a^	NR^a^	steroids	SARS-CoV-2 related facial nerve palsy
**38. Giannantonio S. et al. 2021 Jun.**	Case Report	m	13	fever,asthenia, headache, nausea and dizziness	vertigo, horizontal-torsional grade III nystagmus at Frenzel glass test	NR^a^	BRAIN: normal findings.	steroids	SARS-CoV-2 related cranial neuropathy (VIII cn^c^)
**42. Verkuil LD et al. 2020 Aug.**	Correspondence	f	14	fever, headache, rash, diarrhoea, and dyspnoea	right esotropia, papilledema	increased opening pressure	BRAIN: signs of elevated intracranial pressure	acetazolamide, prednisone	VI cn^c^ involvement in Pseudotumor Cerebri MIS-C related
**43. Baccarella A et al. 2021 Feb.**	Case Report	m	9	fever, headache, abdominal pain	diplopia and right esotropia	normal findings	BRAIN: normal findings	acetazolamide	VI cn^c^ involvement in Pseudotumor Cerebri MIS-C related
		m	6	headache	diplopia, right esotropia and bilateral papilledema	normal findings	BRAIN: signs of elevated intracranial pressure.	steroids	VI cn^c^ involvement in Pseudotumor Cerebri MIS-C related
**47. Botre A et al. 2021 Sep-Oct.**	Case Report	m	6	fever	rapidly progressive ascending paralysis	normal findings	SPINE: contrast enhancement of cauda equina and nerve roots.	IVIG^b^, steroids, plasma exchange	SARS-CoV-2 related GBS^d^
**50. Al Haboob AA et al. 2021 Jul**	Case Report	m	11	vomit, diarrhea, headache	bilateral ptosis, ataxia, generalized tonic-clonic seizures, weak gag reflex, absent cough absent deep tendon reflexes	albuminocytologic dissociation	BRAIN - SPINE: aspecific findings	IVIG^b^, Hydralazine	SARS-CoV-2 related PRES and Miller-Fisher syndrome

m: male; f: female: CSF*: Cerebrospinal fluid (CSF) analysis; MRI: magnetic resonance imaging; NR^a^: not reported in the article; IVIG^b^: Intravenous Immunoglobulin; cn^c^: cranial nerve/nerves.

The number preceding the author refers to the order of the article in the bibliography.

One of the limitations of our observations is that the symptoms of published cases have been attributed to SARS-CoV-2 infection in cases of laboratory evidence of infection, but also in cases of temporal association with flu-like symptomatology or in cases of close contact with affected cases. On the other hand, in the early months of the pandemic, laboratory tests were not readily available. Even to this day, microbiological tests are often negative when neurological symptoms occur, and serological tests are not always performed. Reports of cranial neuropathy have dramatically decreased after the first year of the pandemic. Therefore, we assume that the current variants show less neurotropism or we speculate that in the early period of the pandemic, given the wide circulation of the virus, the scientific world correlated neurological symptoms with the presence of the virus by overestimating the real causality. In conclusion, as clinicians we need to keep in mind the possible links between neurological manifestations and SARS-CoV-2 infection. As clinical researchers, we must make a concerted effort to make a greater contribution to the literature on this topic.

## Author contributions

LP, MF, MC, CG, SG, PI, and AS: conceptualization, data curation, resources, investigation, writing—original draft, methodology, visualization, supervision, and writing—review and editing. All authors contributed to the article and approved the submitted version.

## Funding

All phases of this study were supported by the Department of Mother and Child and Urological Sciences, Sapienza University of Rome, Italy.

## Conflict of interest

The authors declare that the research was conducted in the absence of any commercial or financial relationships that could be construed as a potential conflict of interest.

## Publisher’s note

All claims expressed in this article are solely those of the authors and do not necessarily represent those of their affiliated organizations, or those of the publisher, the editors and the reviewers. Any product that may be evaluated in this article, or claim that may be made by its manufacturer, is not guaranteed or endorsed by the publisher.
